# An examination of behavioural and emotional problems in children exposed prenatally to the 27F Chilean earthquake: findings from the ELPI cohort

**DOI:** 10.1007/s00127-023-02433-z

**Published:** 2023-02-17

**Authors:** María Francisca Morales, Lisa-Christine Girard, Vilas Sawrikar, Angus MacBeth

**Affiliations:** 1 Department of Clinical Psychology, School of Health in Social Science, The University of Edinburgh, Edinburgh, UK; 2 Department of Psychosocial Science, University of Bergen, Bergen, Norway

**Keywords:** Prenatal stress, Natural disasters, Early childhood, Behavioural and emotional problems, Propensity score matching

## Abstract

**Purpose:**

Associations between prenatal earthquake exposure and children’s mental health remain unclear. Moreover, there is a paucity of research using quasi-experimental statistical techniques to diminish potential selection bias. Thus, this study aimed to explore the impact of prenatal exposure to the Chilean earthquake of 2010 on children’s behavioural and emotional problems between 1½ and 3 years old using propensity score matching.

**Methods:**

Participants included 1549 families from the *Encuesta Longitudinal de la Primera Infancia* cohort in Chile. Maternal reports using the Child Behaviour Checklist (CBCL) were used to assess behavioural and emotional problems between 1½ and 3 years old. Information on prenatal earthquake exposure was collected via maternal report. The Kernel matching estimator was used to compare the average treatment effects of children who were exposed to the earthquake compared to those who were not.

**Results:**

Five of the seven CBCL outcomes were statistically significant after matching and adjustment for multiple testing, suggesting greater difficulties for exposed children which included emotional reactivity, anxious/depressed, sleep problems, attention problems, and aggression (mean difference of 0.69, 0.87, 0.73, 0.85, 3.51, respectively). The magnitude of the effect was small to medium.

**Conclusion:**

Findings contribute to the potential causal inferences between prenatal earthquake exposure and increased behavioural and emotional problems in early childhood. Results suggest that in utero experiences may have long-term consequences for infants’ well-being, supporting the need for specific interventions in pregnancy after natural disasters.

**Supplementary Information:**

The online version contains supplementary material available at 10.1007/s00127-023-02433-z.

## Introduction

Behavioural and emotional problems in childhood are associated with increased risk for several adverse outcomes across the lifespan, such as academic failure, substance abuse and depression, among others [1, 2]. Consequently, particular attention is placed on studying risk factors to better understand aetiology and inform prevention strategies. In the study of risk factors, several antecedents at the perinatal (e.g. prematurity, low birth weight, incubator staying, breastfeeding, maternal mental health) [3–6], child (e.g. sex, education attendance) [7, 8], and family levels (e.g. single parent, maternal age, educational level, occupation, socioeconomic status) [9–11] have been found to be associated with child behavioural and emotional problems. Among these factors, there is an increasing interest in examining how prenatal stress (e.g. depression, anxiety, stressful life events exposure) can increase the risk for children’s behavioural and emotional problems such as aggression, depression and anxiety [12, 13].

Studies exploring the effects of prenatal stress face the challenge of disentangling the extent to which behavioural and emotional problems are associated with the independent impact of prenatal stress versus other confounding factors. More precisely, these studies confront the selection bias problem, where children who experienced prenatal stress may differ from their counterparts for reasons other than stress exposure. Because randomisation of participants is unfeasible in this field, other alternatives such as quasi-experimental designs have been proposed to diminish selection bias. Quasi-experimental designs help to mimic randomisation by creating ‘treated’ and ‘control’ groups that are similar in observed characteristics on average across these conditions (i.e. experience of prenatal stress or not), providing more reliable estimates than when using observational studies [14]. Consequently, various observational studies nowadays employ quasi-experimental approaches [e.g. 3, 15, 16] including propensity score matching (PSM), instrument variables and natural experiments, with an increased interest in examining natural disasters for assessing prenatal stress.

Natural disasters create disruptions in the population across several dimensions and are considered a public health concern [17, 18]. As a result of their unanticipated characteristics, natural disasters may be classified as quasi-experimental designs [19]. When a pregnant mother is exposed to a natural disaster, the experienced stress is independent of other confounding factors, such as genetic inheritance or maternal behaviours, helping to circumvent the obstacles of selection bias. Consequently, several studies in the last decade have inspected the effects of prenatal natural disaster exposure as a measure of stress, reporting positive associations with children’s behavioural and emotional problems [e.g. 15, 20–23]. Furthermore, a recent meta-analysis synthesised the associations of prenatal exposure to natural disasters and children’s outcomes, reporting a positive statistically significant effect on children’s behavioural and emotional problems [24]. However, this meta-analysis also exposed limitations in its included studies, such as small sample sizes, unrepresentative demographics (e.g. higher SES), and no studies inspecting earthquake exposure when assessing behavioural and emotional problems. Given the prevalence of earthquakes worldwide (i.e. 20,000 earthquakes around the globe per year) [25], examining this type of natural disaster may better inform public health initiatives to mitigate the adverse effects of disruptive natural disasters in the population, particularly children. Moreover, none of the included studies used quasi-experimental statistical techniques (e.g. PSM) to overcome further potential selection bias.

### Aims

More well-controlled research using natural experiments and quasi-experimental statistical techniques is needed to improve our understanding of the potential impact of prenatal exposure to natural disasters on children’s outcomes. Accordingly, the current study aimed to explore the effect of earthquake exposure prenatally on children’s behavioural and emotional problems between 1½ and 3 years old by comparing exposed children to children of the same age who were not exposed to the earthquake in utero or thereafter using PSM. More precisely, this study used the Chilean earthquake of 2010, the sixth-largest earthquake in the world recorded by a seismograph. Children’s ages were chosen considering the importance of examining infant mental health to inform early prevention strategies and enhance the child’s well-being [26]. In line with previous findings [15, 20–24], it was hypothesised that children who were exposed to the earthquake prenatally (i.e. the ‘treatment’ group) would display increased behavioural and emotional problems in early childhood compared to children who were not exposed to the earthquake in utero or thereafter (i.e. control group). Following reporting conventions in the quasi-experimental literature, the terms treatment and control groups are used henceforth.

## Methods

### Participants

**Fig. 1:**
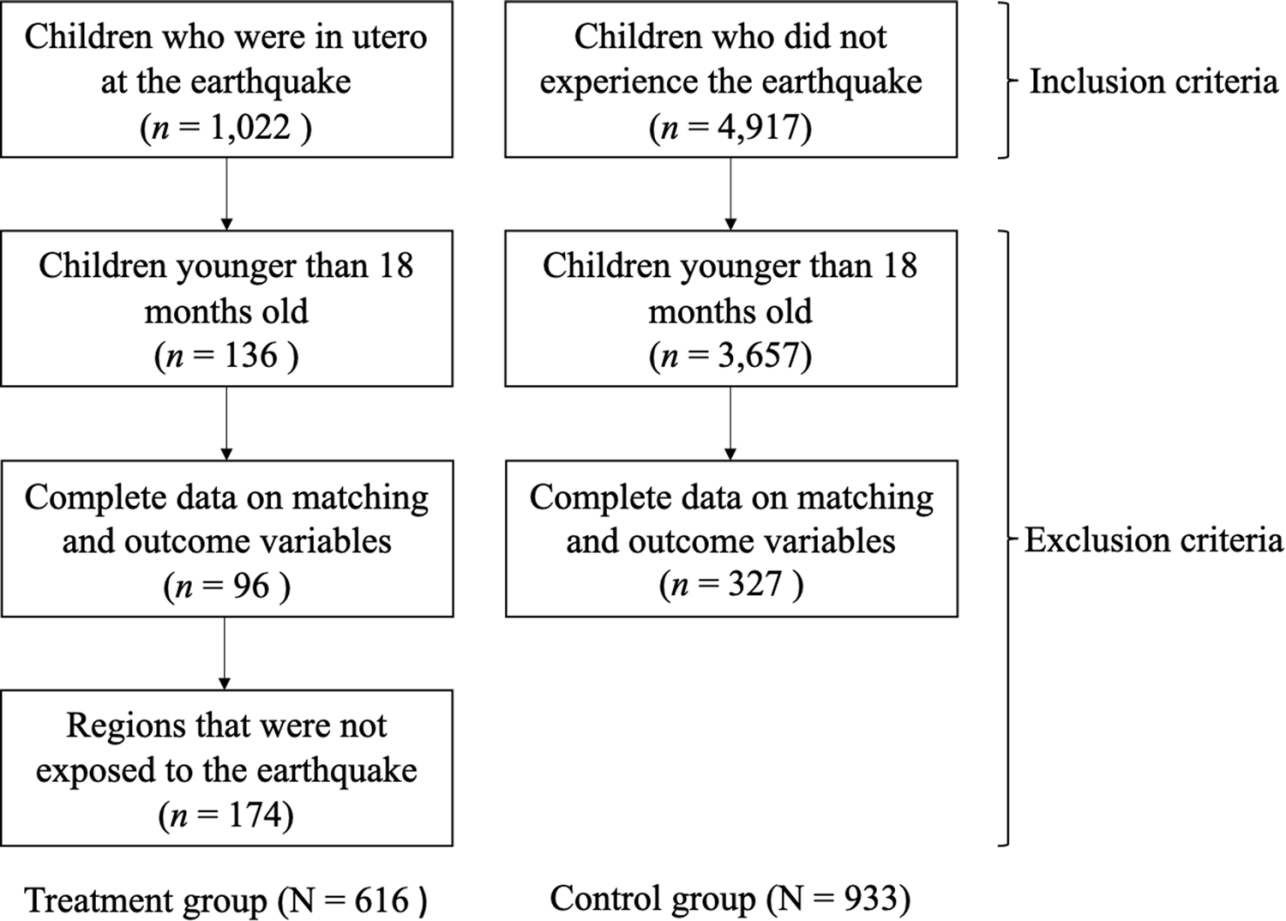
Study group selection

Participants included families enrolled in the *Encuesta Longitudinal de la Primera Infancia* (ELPI—Longitudinal Survey of Early Childhood) study, a representative cohort of Chilean families conducted between 2010 and 2017. For complete details of the ELPI study design, see Behrman [27]. The inclusion criterion for the treatment group was children in utero during the Chilean earthquake of February 2010. Mothers were retrospectively asked: ‘Were you pregnant with the selected child at the time of the earthquake on the 27th of February 2010? (yes/no)’. These children were included in the supplementary sample of wave 2 (*n* = 3135), with 1022 of them being in utero during the earthquake. For the control group, the inclusion criterion was children who did not experience the 2010 earthquake in utero or after they were born, including their aftershocks. Thus, the supplementary sample from wave 3 was added (*n* = 4917). Exclusion criteria were children’s age since the selected outcome assessment (i.e. CBCL 1½-5) starts from 18 months old, excluding children under this age (treatment group = 136; control group = 3657). Children who did not have complete data on the matching variables and the outcome assessment were also excluded (treatment group = 96; control group = 327) since both are conditions required for the proper use of PSM. Finally, children from the treatment group who were not in one of the six regions affected by the earthquake (i.e. Valparaíso, Metropolitana, O’Higgins, Maule, Bío-Bío, Araucanía) were excluded from the sample (treatment group = 174). Consequently, the final sample used in the current study included 616 children in the treatment group and 933 children in the control group, comprising a total of 1549 children aged between 18 and 35 months old. See Fig. 1 for a graphical representation of groups’ selection. Characteristics of the included sample at the perinatal, child and family levels prior to matching can be found in Table 1. Ethical approval of the ELPI cohort was granted by the Microdata Centre of the University of Chile (Centro Microdatos, Universidad de Chile).

**Table 1:** Pre-matching characteristics of the included sample at the perinatal, child and family levels

	Treatment group *n* (%)	Control group *n* (%)	*p*
Perinatal level			
Prematurity (yes)	44 (7.1%)	187 (20.0%)	≤ 0.001
Low birth weight (yes)	22 (3.6%)	67 (7.2%)	0.003
Incubator (yes)	27 (4.4%)	74 (7.9%)	0.006
Breastfeeding length			
Never	146 (23.7%)	63 (6.8%)	≤ 0.001
Up to 6 months	177 (28.7%)	277 (29.7%)	
Between 7 and 12 months	166 (27.0%)	212 (22.7%)	
13 months or more	127 (20.6%)	388 (40.8%)	
Antenatal depression (yes)	59 (9.6%)	75 (8.0%)	0.292
Postnatal depression (yes)	77 (12.5%)	130 (13.9%)	0.417
Child level			
Child sex (male)	306 (49.7%)	482 (51.7%)	0.444
Child attendance to educational system (no)	440 (71.4%)	579 (62.1%)	≤ 0.001
Family level			
Maternal age			
≤ 24 years old	197 (32.0%)	223 (23.9%)	0.006
25–29 years old	131 (21.3%)	219 (23.5%)	
30–34 years old	139 (22.6%)	245 (26.3%)	
≥ 35 years old	149 (24.2%)	246 (26.4%)	
Single parent (yes)	184 (29.9%)	369 (39.6%)	≤ 0.001
Maternal education			
No formal education	1 (0.2%)	4 (0.4%)	≤ 0.001
Primary complete	95 (15.4%)	62 (6.7%)	
Secondary complete	406 (65.9%)	464 (49.7%)	
Vocational training	58 (9.4%)	161 (17.3%)	
University training	56 (9.1%)	242 (25.9%)	
Maternal occupation (unemployed)	15 (2.4%)	66 (7.1%)	≤ 0.001
Provisional health system (public)	550 (89.3%)	813 (87.1%)	0.203

Treatment group = children who were in utero at the 27F earthquake (*n* = 616), control group = children conceived after the 27F earthquake, who have not experienced the earthquake in utero or after (*n* = 933). Low birth weight < 2.500 gr. Prematurity = under 37 gestational weeks. Attendance to educational system = any educational establishment (e.g. nursery, preschool)

### Measurement

Children’s behavioural and emotional problems were assessed using maternal reports of the Child Behaviour Checklist preschool version (CBCL 1½-5) [28]. The CBCL 1½-5 is a screening tool assessing multiple behavioural and emotional difficulties in children aged 1½ to 5 years old. This version comprises 7 subscales: emotionally reactive (9 items), anxious/depressed (8 items), somatic complaints (11 items), withdrawn (8 items), sleep problems (7 items), attention problems (5 items) and aggression (19 items). Items are rated on a three-point Likert scale ranging from 0 (*not true*) to 2 (*very or often true*), with higher scores indicating more problematic behaviours. The CBCL 1½–5 has a Spanish version and has been previously validated with Chilean samples, reporting good psychometric properties with Cronbach’s alpha coefficients of 0.88 for the internalising and 0.90 for the externalising scales [29].

Several variables associated with children’s behavioural and emotional problems were considered to match treatment and control groups. According to previously reported associations and data availability in both supplementary samples, 13 variables at the perinatal, child and family levels were included to match groups. At the perinatal level, mothers reported retrospectively on perinatal and birth outcomes, which included: prematurity (categorised as under 37 gestational weeks; yes/no), low birth weight (categorised as under 2.500 gr.; yes/no), stay in an incubator after delivery (yes/no), breastfeeding (never, up to 6 months, between 7 and 12 months, 13 months or more), antenatal depression (yes/no) and postnatal depression (yes/no). At the child level, sex (male/female) and attendance at any educational system level (e.g. nursery, preschool; yes/no) were included as confounders. Lastly, at the family level, maternal age (≤ 24, 25–29, 30–34, ≥ 35), single parent (yes/no), maternal education (no formal education/ primary complete/ secondary complete/ vocational training/ university training), maternal occupational status (employed/unemployed) and health provisional system (public/private) were included. The Chilean health provisional system (public/private) was used as a proxy for socioeconomic status. The private system in Chile is associated with more health resource access and higher economic wealth, whilst the public system is associated with a lower family income [30].

### Statistical analysis

**Fig. 2:**
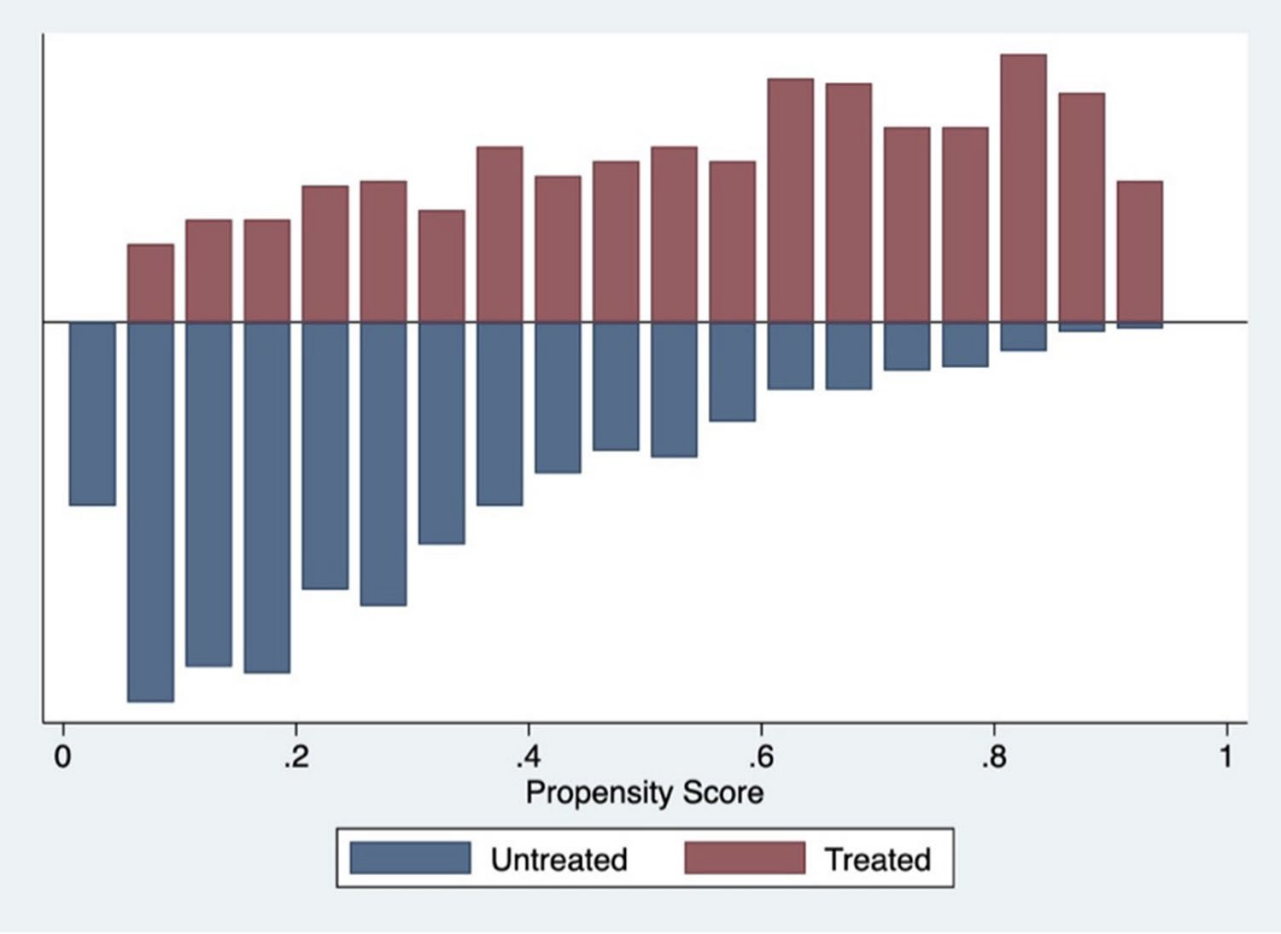
Overlapping support: distribution of propensity scores

**Fig. 3:**
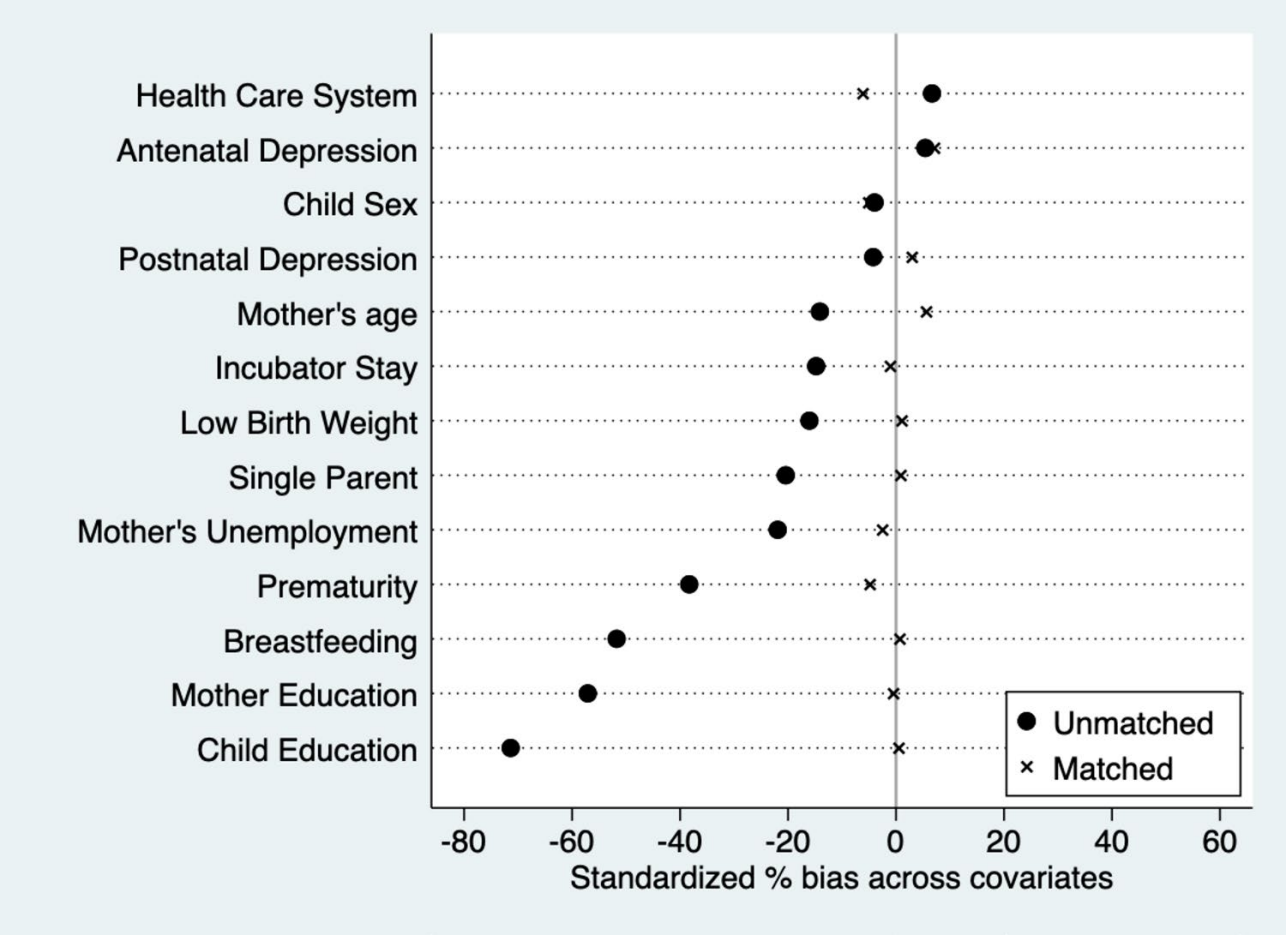
Standardised differences across covariates: pre-matching and post-matching

This study employed PSM to reduce selection bias diminishing treatment and control groups differences by matching groups on relevant observable factors. We used the Kernel estimator without common support to match participants. Kernel matching is a non-parametric matching estimator that, after randomly ordering all participants, matches each treatment participant to all control participants with greater weight given to those with more similar propensity scores [14]. One advantage of this approach is the lower variance achieved because more information is used, while a limitation is that possible used observations are bad matches. However, the results with the Kernel estimator were consistent across different matching options (i.e. nearest neighbour with and without replacement and Kernel with common support), providing further support for this decision (see supplementary file I). Thereafter, common support area and balance checks were inspected on individual matching variables and the overall model to assess the quality of matching. All sampled children fell within the common support area as can be seen by the distributional balance on the distance measure of the propensity score across participants (Fig. 2). Balance checks of individual variables revealed no statistically significant differences between groups after matching (Table 2) and exhibited considerable bias reduction between matched groups on all the matching variables (i.e. 0.5–7.1%), with 3.0% of mean remaining bias for the overall model (Fig. 3). Rosenbaum and Rubin [31] suggested that less than 20% remaining bias denotes good matching, indicating that the matching strategy implemented in this study was successful. The Results section reports the average treatment effects of treated participants (ATT), which is the effect on the population potentially exposed to treatment compared to their not treated counterparts. Statistical significance levels were adjusted for multiple testing using the Bonferroni correction method. The term significant is used hereafter to denote statistical significance. All analyses were conducted using Stata V.17 software.

**Table 2:** Matched and unmatched covariates differences

	Matched		Unmatched	
	t	*p*	t	*p*
Prematurity	− 1.06	0.290	** − 7.08**	** ≤ 0.001**
Low birth weight	0.23	0.819	** − 2.99**	**0.003**
Incubator	** − **0.22	0.826	** − 2.77**	**0.006**
Breastfeeding length	0.12	0.906	** − 10.05**	** ≤ 0.001**
Antenatal depression	1.26	0.209	1.05	0.292
Postnatal depression	0.54	0.590	** − **0.81	0.417
Child sex	** − **0.90	0.369	** − **0.76	0.444
Child attendance to educational system	0.09	0.928	** − 13.65**	** ≤ 0.001**
Maternal age	0.99	0.323	** − 2.73**	**0.006**
Single parent	0.15	0.877	** − 3.91**	** ≤ 0.001**
Maternal education	** − **0.09	0.925	** − 10.78**	** ≤ 0.001**
Maternal occupation	** − **0.58	0.563	** − 4.03**	** ≤ 0.001**
Provisional health system	** − **1.18	0.240	1.27	0.203

All significant differences (i.e. *p* < 0.05) are highlighted in bold

## Results

Prior to matching, comparisons of children who never experienced an earthquake to those in utero during the 2010 earthquake showed significant differences on all CBCL subscales except withdrawn. However, only five subscales remained significant after matching, including emotionally reactive, anxious/depressed, sleep problems, attention problems and aggression. Results remained significant after controlling the alpha level for multiple testing. Differences between treatment and control groups in children from 1½ to 3 years old revealed that children in utero during the earthquake displayed higher scores on all the problematic behaviours, with mean differences showing small to medium effect sizes. Results can be found in Table 3.

**Table 3:** Pre-matching and post-matching results on children’s behavioural and emotional problems

	Pre-matching				Post-matching				*d*
	T	C	Diff	SE	T	C	Diff	SE	
Emotionally reactive	2.64	1.81	0.83*	0.13	2.64	1.94	0.69*	0.19	0.29
Anxious/depressed	3.87	2.94	0.93*	0.13	3.87	3.00	0.87*	0.20	0.35
Somatic complaints	2.63	2.30	0.33*	0.12	2.63	2.52	0.11	0.18	0.05
Withdrawn	2.49	2.34	0.15	0.11	2.49	2.38	0.11	0.17	0.05
Sleep problems	2.77	2.29	0.49*	0.12	2.77	2.04	0.73*	0.19	0.31
Attention problems	3.58	2.79	0.79*	0.10	3.58	2.73	0.85*	0.15	0.46
Aggression	13.81	9.98	3.83*	0.38	13.81	10.30	3.51*	0.57	0.48

^*^Denotes significance at the corrected significance level. All significant differences are highlighted in bold. T refers to the treatment group (children who were in utero during the 27F earthquake, *n* = 616) and C refers to the control group (children conceived after the 27F earthquake, who have not experienced the earthquake in utero or after, *n* = 933). Diff denotes the differences between groups in scores. SE represents the standard errors. *d* refers to Cohen’s d effect size

## Discussion

Using a large population cohort from Chile with a natural disaster as a quasi-experimental design and PSM as a quasi-experimental statistical technique, findings suggest that earthquake exposure prenatally is associated with higher behavioural and emotional problems in early childhood. This study adds novel findings to the literature inspecting the associations between prenatal stress induced by natural disasters by (i) examining an earthquake as an environmental stressor since they have been mostly overlooked in this field, and (ii) being the first study to our knowledge that implements PSM to minimise differences between groups on observable characteristics. Therefore, study findings provided further evidence around the ‘causal effect’ of prenatal stress following natural disasters on behavioural and emotional outcomes with methodological improvements using PSM.

The study hypothesised that children prenatally exposed to the 2010 Chilean earthquake would display increased behavioural and emotional problems in early childhood. This prediction was largely supported since, after the matching analysis, five of the seven inspected outcomes were significant, including emotionally reactive, anxious/depressed, sleep, attention and aggressive problems. These findings are in line with previous research inspecting natural disasters [24], suggesting a positive association between prenatal stress exposure and children’s internalising scores [15, 20, 21], aggression and total externalising scores [20, 32, 33], and sleep problems [23]. Conversely, findings challenge Berthelon and colleagues [34] reported results, who only described associations for attentional problems using the same Chilean cohort as this study. However, they included children conceived shortly after the 2010 earthquake for the control group, and those pregnant women were still exposed to the effects of the aftershocks and potentially experienced the earthquake’s negative psychological and social consequences. Thus, methodological differences in their control group may explain the disparities in the results of emotionally reactive, anxious/depressed, sleep and aggressive problems.

Regarding the practical importance of these associations, the magnitude of the effect of being in utero during the earthquake for each outcome was found to be small to medium, with larger effects for aggression and attention problems, followed by anxious/depressed symptoms, sleep and emotionally reactivity. Even though effect sizes were found to be small to medium in all the significant outcomes, they still have relevant practical implications, especially considering that a small to moderate increase in children’s emotionally reactive, anxious/depressed, sleep, attention and aggressive problems may increment the public health burden associated with behavioural and emotional problems [26]. Compared to the pooled effect size reported by the meta-analyses of Lafortune and colleagues [24], the effect magnitudes from this study were larger than the effect sizes described for flood disasters (*r* = 0.08) and similar to ice storms (*r* = 0.26). These findings may reflect the disparities in strength and duration of each natural disaster and their potential effects on the foetus, suggesting a dose–response association where the greater the strength of the natural disaster, the larger the effects are on children’s behavioural and emotional outcomes. For example, the Chilean earthquake of 2010 was particularly strong and provoked several damages and distress in the population [35]. Hence, our results emphasise the importance of continuing exanimating earthquakes as environmental stressors for pregnancy since their associations with children’s behavioural and emotional outcomes may be larger than for other natural disasters.

An evolutionary perspective may help explain these results [13, 36]. The concept of predictive adaptive response [13, 37] explains that early experiences in utero are a source of information about the environment in which the child will live and that foetal development is an opportunity to adapt to that future environment and have a successful development. Therefore, children’s emotional reactivity, anxiety/depression, sleep, attention and aggression problems potentially reflect adaptative behaviours to predicted stressful environments [37]. These predictive adaptative responses have an evolutionary value when the postnatal environment is perceived as dangerous, and behavioural and emotional outcomes help ensure survival in threatening environments [13]. For example, emotionally reactive symptoms may incite caregiver attention and boost survival chances, depressive symptoms may provoke avoidance of threatening situations, sleep and attention problems can potentially increase extra vigilance and help to identify threats in the environment, whereas aggressive behaviours may help to provoke a defensive reaction against external threats [36, 38]. Accordingly, from an evolutionary perspective, it can be presumed that emotional outcomes that were not significant after the matching and adjustment procedures (i.e. somatic complaints and withdrawal) are not effective resources to increase survival against external threats. Hence, emotional reactivity, anxiety/depression, sleep, attention and aggression problems are potentially more efficient adaptative strategies that help to avoid external threats and/or increase caregivers’ contingent interaction processes. However, all these behaviours that may be adaptative in an environment with potential physical danger can be problematic when a mismatch exists between what was predicted in the prenatal period and the later environment [13]. Therefore, those adaptative behaviours can hamper children from achieving the demands of the modern world and be understood as maladaptive behaviours that affect children’s adjustment and well-being.

This study has several strengths, including using a national cohort from Chile with a larger sample size compared to other studies inspecting natural disasters. Moreover, this study used a natural disaster that has been frequently overlooked and a PSM technique with several matching confounders associated with children’s behavioural and emotional outcomes. Nevertheless, some limitations need consideration. Despite the benefits of using a quasi-experimental statistical technique, there may still be some sources of selection bias. While PSM is a technique designed to diminish selection bias, the statistical approach can only match groups on observable characteristics whilst unobservable variables that may impact children’s outcomes are not considered [31]. Moreover, only variables available in the two waves of the ELPI dataset were used, potentially omitting other important characteristics (e.g. attachment and social support). Therefore, important observable and unobservable variables may still contribute to the reported associations, and any causal inference of results warrants caution. Second, mothers’ reports were used to gather information on both children’s outcomes and matching variables. Therefore, shared method variance is a potential concern, and results should be interpreted with caution. Future studies could include multiple informants of children’s behaviours (e.g. teachers) to mitigate this concern. Third, since waves from different years were used for the treatment (wave 2) and control (wave 3) groups, there is a potential unobserved cohort effect in operation besides the earthquake impact. However, considering that children included in the third wave were the only ones in the ELPI cohort who were utterly unaffected by the 2010 earthquake (they were born after the earthquake), the choice of having them as the control group was made to ensure no indirect exposure effects that could be attributed to the earthquake. Finally, this study would have been an ideal alternative to assess timing of prenatal stress using earthquake exposure. Unfortunately, the ELPI dataset did not have information on the week of pregnancy when the natural disaster occurred, and the date of birth was unavailable for around 40% of children in the treatment group, making it unfeasible to include timing in these results. Therefore, future studies inspecting the effect of natural disasters should ask the specific week of pregnancy at the event or date of birth with associated gestational weeks. Additionally, the study would have also been a good option to assess potential mediating pathways (e.g. financial losses and household damage) between natural disaster in utero exposure and children’s behaviours. However, none of the included participants had available information on those variables. Therefore, future studies should further explore specific mediating pathways to unpack how the risk of being in utero during a natural disaster is transmitted to children’s behavioural and emotional problems.

Despite the limitations and considering the strengths of this study, these findings have important practical implications for regions susceptible to experiencing earthquakes. This study’s findings support that prenatal natural disaster exposure is associated with later adverse consequences on children’s behavioural and emotional outcomes. Nonetheless, prenatal stress effects do not have to be irreversible since the postnatal environment can buffer them [39]. Therefore, it is important to develop early interventions at the policy and clinical levels to mitigate the adverse effects of prenatal stress following natural disasters. For example, it has been emphasised that mental health services such as psychological first aid should be widely offered in regions affected by natural disasters and not wait until people ask for support [17], with available guidelines for natural disasters created by the World Health Organization and War Trauma Foundation and World Vision International [40]. Moreover, further strategies should be implemented in addition to the ones proposed immediately after the event during pregnancy. For instance, postnatal interventions promoting secure attachment styles and increasing parental social support could help to buffer the adverse effects of prenatal stress on children’s development [39].

## Supplementary Information

Below is the link to the electronic supplementary material.

Supplementary file1 (PDF 736 KB)

## Data Availability

The datasets generated and analysed during the current study are available in the Ministerio de Desarrollo Social y Familia repository, http://observatorio.ministeriodesarrollosocial.gob.cl/elpi-primera-ronda.
