# Supplementary material for: An examination of behavioural and emotional problems in children exposed prenatally to the 27F Chilean earthquake: findings from the ELPI cohort
Source: Soc Psychiatry Psychiatr Epidemiol. 2023 Feb 17;58(7):1065–73. doi: 10.1007/s00127-023-02433-z (PMC10261207; doi:10.1007/s00127-023-02433-z)
Supplement: Supplementary file 1 — Supplementary file1 (PDF 736 KB) [file 127_2023_2433_MOESM1_ESM.pdf]

Article title: An examination of behavioural and emotional problems in children exposed prenatally to the 27F Chilean earthquake: Findings from the ELPI cohort

Journal name: Social Psychiatry and Psychiatric Epidemiology

Author names: María Francisca Morales[a] (ORCID: 0000-0002-1971-8085), Lisa-Christine Girard[b] (ORCID: 0000-0003-3817-034X), Vilas Sawrikar[a] (ORCID: 0000-0001-8160-0232), Angus MacBeth[a] (ORCID: 0000-0002-0618-044X).

Affiliations: [a]Department of Clinical Psychology, School of Health in Social Science, The University of Edinburgh, United Kingdom. [b]Department of Psychosocial Science, University of Bergen, Norway.

Corresponding author: María Francisca Morales, mariafrancisca.morales@ed.ac.uk

Supplementary file I: Matching Algorithms

Nearest neighbour with replacement

Table S1: Common support using nearest neighbour with replacement

Treatment assignment	Off support	On support	Total
Untreated	0	933	933
Treated	0	616	616
Total	0	1,549	1,549

Figure S1: Overlapping Support: Distribution of Propensity Scores using nearest neighbour with replacement

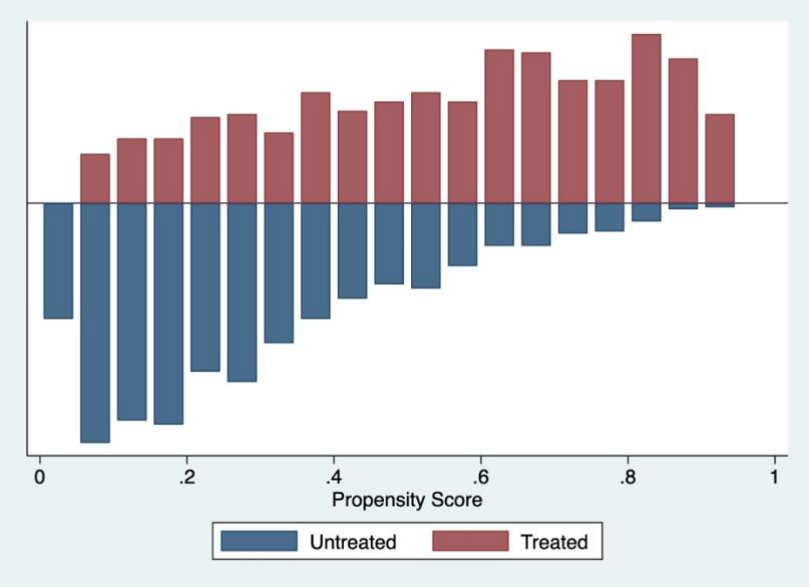

Table S2: Standardised Differences across Covariates: Pre-Matching and Post-Matching using nearest neighbour with replacement

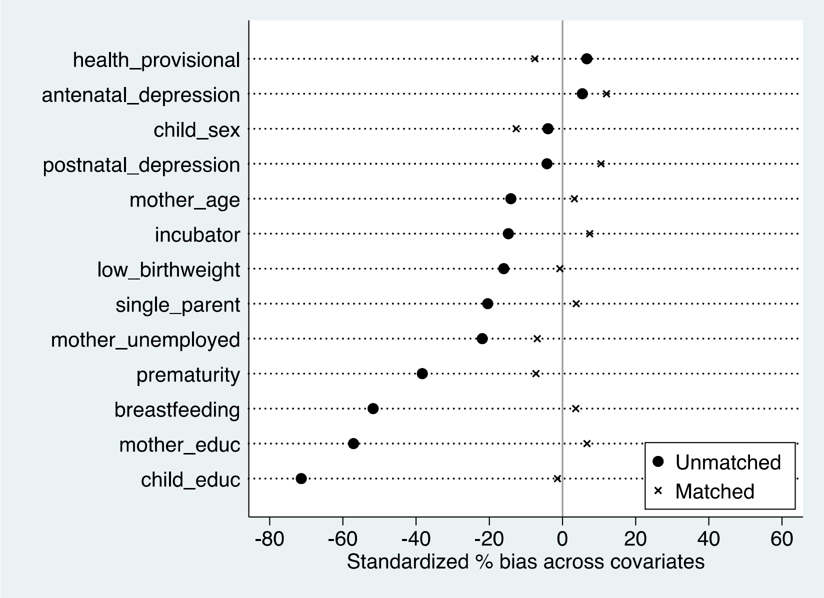

Table S2: Pre-matching and post-matching results on children's behavioural and emotional problems using nearest neighbour with replacement

	Pre-matching					Post-matching				
	T	C	Diff	SE	T-stat	T	C	Diff	SE	T-stat
Emotionally reactive	2.64	1.81	0.83	0.13	6.62	2.64	1.79	0.85	0.24	3.52
Anxious/depressed	3.87	2.94	0.93	0.13	7.19	3.87	2.83	1.04	0.26	3.93
Somatic complaints	2.63	2.30	0.33	0.12	2.78	2.63	2.59	0.04	0.25	0.14
Withdrawn	2.49	2.34	0.15	0.11	1.38	2.49	2.29	0.19	0.24	0.81
Sleep problems	2.77	2.29	0.49	0.12	3.97	2.77	2.05	0.72	0.24	3.02
Attention problems	3.58	2.79	0.79	0.10	8.24	3.58	2.76	0.82	0.19	4.36
Aggression	13.81	9.98	3.83	0.38	10.03	13.81	10.19	3.62	0.78	4.61

Nearest neighbour without replacement

Table S3: Common support using nearest neighbour without replacement

Treatment assignment	Off support	On support	Total
Untreated	0	933	933
Treated	232	384	616
Total	232	1,317	1,549

Figure S3: Overlapping Support: Distribution of Propensity Scores using nearest neighbour without replacement

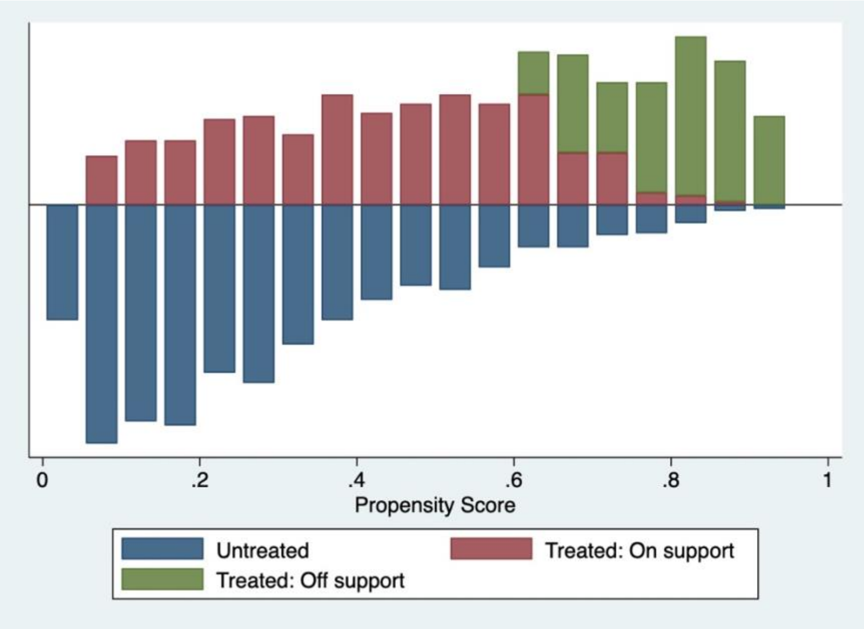

Figure S4: Standardised Differences across Covariates: Pre-Matching and Post-Matching using nearest neighbour without replacement

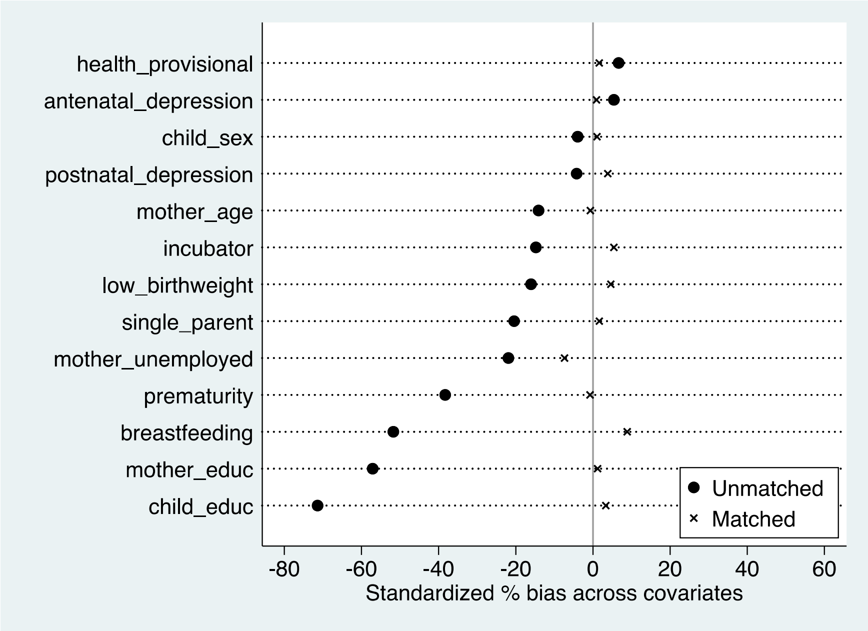

Table S4: Pre-matching and post-matching results on children's behavioural and emotional problems using nearest neighbour without replacement

	Pre-matching					Post-matching				
	T	C	Diff	SE	T-stat	T	C	Diff	SE	T-stat
Emotionally reactive	2.64	1.81	0.83	0.13	6.62	2.43	1.90	0.53	0.18	2.99
Anxious/depressed	3.87	2.94	0.93	0.13	7.19	3.67	3.01	0.67	0.19	3.55

Somatic complaints	2.63	2.30	0.33	0.12	2.78	2.46	2.41	0.05	0.17	0.30
Withdrawn	2.49	2.34	0.15	0.11	1.38	2.38	2.55	-0.17	0.15	-1.11
Sleep problems	2.77	2.29	0.49	0.12	3.97	2.70	2.22	0.48	0.18	2.72
Attention problems	3.58	2.79	0.79	0.10	8.24	3.47	2.82	0.65	0.14	4.79
Aggression	13.81	9.98	3.83	0.38	10.03	13.31	10.35	2.96	0.56	5.32

Kernel with common support

Table S5: Common support using Kernel with common support

Treatment assignment	Off support	On support	Total
Untreated	0	933	933
Treated	8	608	616
Total	8	1,541	1,549

Figure S5: Overlapping Support: Distribution of Propensity Scores using Kernel with common support

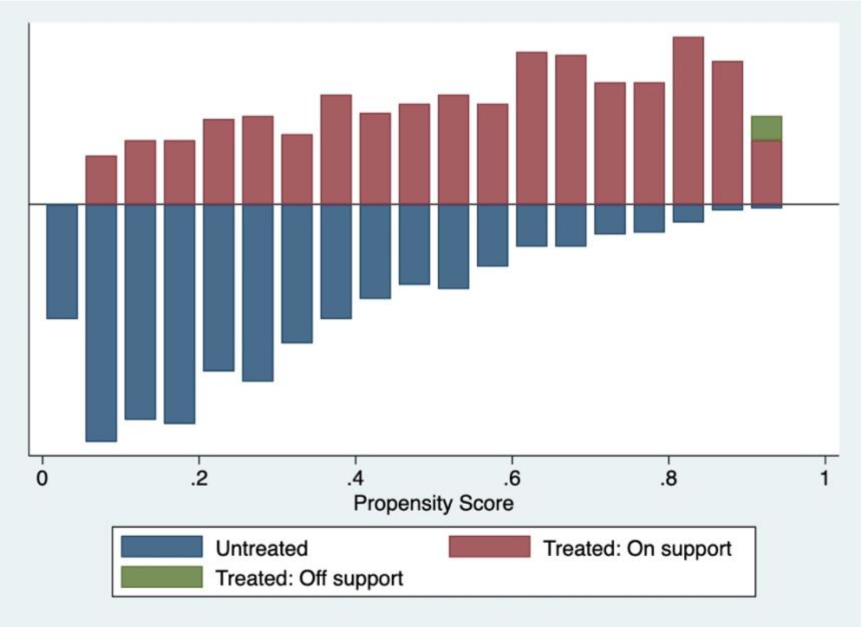

Figure S6: Standardised Differences across Covariates: Pre-Matching and Post-Matching using Kernel with common support

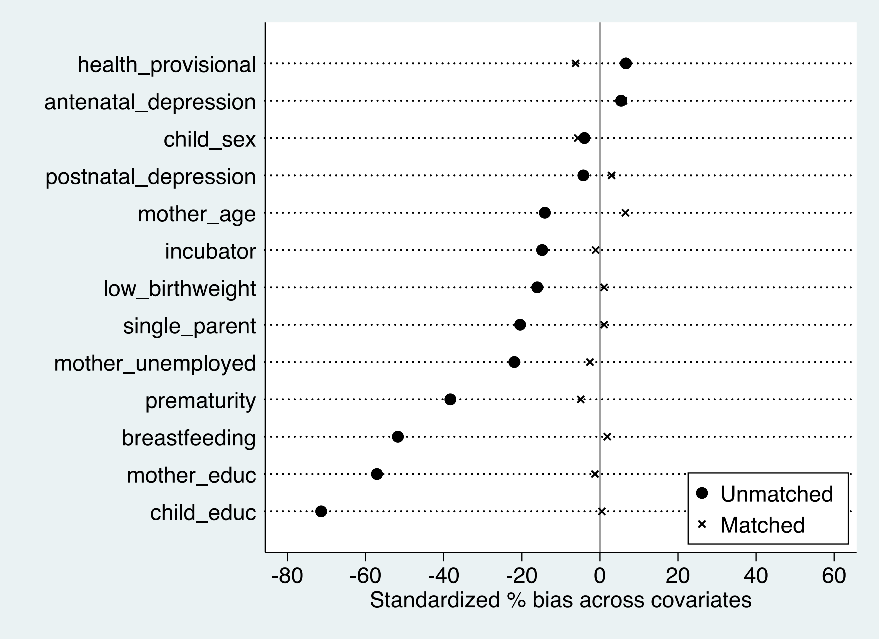

Table S6: Pre-matching and post-matching results on children's behavioural and emotional problems using Kernel with common support

	Pre-matching					Post-matching				
	T	C	Diff	SE	T-stat	T	C	Diff	SE	T-stat
Emotionally reactive	2.64	1.81	0.83	0.13	6.62	2.64	1.95	0.69	0.18	3.76
Anxious/depressed	3.87	2.94	0.93	0.13	7.19	3.86	3.02	0.84	0.19	4.41
Somatic complaints	2.63	2.30	0.33	0.12	2.78	2.62	2.52	0.10	0.18	0.57
Withdrawn	2.49	2.34	0.15	0.11	1.38	2.49	2.39	0.10	0.17	0.58
Sleep problems	2.77	2.29	0.49	0.12	3.97	2.79	2.06	0.73	0.18	3.99
Attention problems	3.58	2.79	0.79	0.10	8.24	3.59	2.75	0.83	0.14	5.86
Aggression	13.81	9.98	3.83	0.38	10.03	13.86	10.33	3.54	0.56	6.34
